# Hormone receptor-positive, HER2-negative, metastatic breast cancer responded well to abemaciclib and exemestane after palbociclib and fulvestrant failure: A case report and literature review

**DOI:** 10.3389/fonc.2022.1022913

**Published:** 2023-01-09

**Authors:** Yan Mao, Meng Lv, Yongmei Wang, Weihong Cao, Wenfeng Li

**Affiliations:** Breast Disease Center, The Affiliated Hospital of Qingdao University, Qingdao, Shandong, China

**Keywords:** CDK4/6 inhibitor, breast cancer, metastatic, abemaciclib, case report

## Abstract

There is uncertainty regarding the usefulness of CDK4/6-inhibitor-based therapy for hormone receptor positive (HR+), human epidermal grow factor receptor 2 negative (HER2−), metastatic breast cancer (MBC), when CDK4/6 inhibitor treatment had previously failed. Furthermore, a biomarker for abemaciclib resistance has not been identified. Herein, we reported outcomes for an HR+/HER2− MBC patient diagnosed with multiple myeloma and treated with abemaciclib and exemestane, who had cancer progression after treatment with palbociclib and fulvestrant. Thalidomide was used in conjunction with all treatments. The patient had a good response to abemaciclib and exemestane, with progression-free survival much longer than previously reported. PIK3CA and TP53 mutations were identified after cancer progression following abemaciclib treatment. It is unclear whether thalidomide increased the effectiveness of abemaciclib. Whether benefit can be derived by the use of PI3K inhibitors, after cancer progression, requires further investigation, and this may be best accomplished by the use of next-generation sequencing.

## Introduction

For hormone receptor-positive (HR+), human epidermal grow factor receptor 2-negative (HER2−), metastatic breast cancer (MBC) patients, endocrine therapy is the first line of treatment, except for patients in visceral crisis (1). Based on multiple trials of cyclin-dependent kinase 4 (CDK4) and CDK6 inhibitors, the combination of a CDK4/6 inhibitor with endocrine therapy has improved the survival and the quality of life for HR+/HER2− MBC patients, with outcomes far better than those with chemotherapy alone (2–8). Therefore, guidelines and consensus recommend a CDK4/6 inhibitor combined with endocrine therapy as the treatment of choice for HR+/HER2− MBC patients. There are three CDK4/6 inhibitors (palbociclib, abemaciclib, and ribociclib) that have been approved by the Food and Drug Administration (FDA) for MBC (2–8), with abemaciclib also approved for adjuvant therapy of HR+/HER2− high risk early breast cancer (9). This is good news for breast cancer patients, but a challenge for oncologists. For example, if an HR+/HER2− MBC patient chose a combination of a CDK4/6 inhibitor with endocrine therapy as a first-line treatment, what would be the best choice as a second line, chemotherapy or another CDK4/6 inhibitor? Which CDK4/6 inhibitor is the best choice? Limited data are available to address these questions. Professor Angela DeMichele provided three endocrine choices at the 2018 ASCO Annual Meeting: change treatment to another CDK4/6 inhibitor, use a different endocrine drug, or add another target drug (e.g., the mTOR inhibitor, everolimus). A retrospective multicenter study found abemaciclib to be well tolerated after prior treatment with palbociclib, with median progression-free survival (PFS) of 5.3 months. These results were similar to the results of the MONARCH-1 study (10). In that study, median PFS was similar for patients who received abemaciclib monotherapy or abemaciclib combined with endocrine therapy. The median PFS was longer (8.4 months, 95% CI, 4.1–NR) for patients who received sequential CDK4/6 inhibitor therapies than for patients who received non-sequential abemaciclib therapy (3.9 months, 95% CI, 2.9–5.7, p=0.0013) (10). As such, some patients may benefit from treatment with another CDK4/6 inhibitor. However, the question is which treatment regimen is the best choice for patients who progressed after prior CDK4/6 inhibitor treatment (i.e., chemotherapy, another CDK4/6 inhibitor, or alternative endocrine therapy)?

Herein, we reported outcomes for one HR+/HER2− MBC patient who was also diagnosed with multiple myeloma. The patient had a good response to non-sequential abemaciclib and exemestane after failure with palbociclib and fulvestrant. Next-generation sequencing (NGS) of circulating tumor DNA (ctDNA), after progression with abemaciclib, identified PIK3CA and TP53 mutations, which suggested that this patient may be sensitive to PI3K inhibitors. This case report provided new insight into a treatment strategy for HR+/HER2− MBC patients after prior failure of combined CDK4/6 inhibitor and endocrine therapy.

## Case presentation

**Figure 1:**
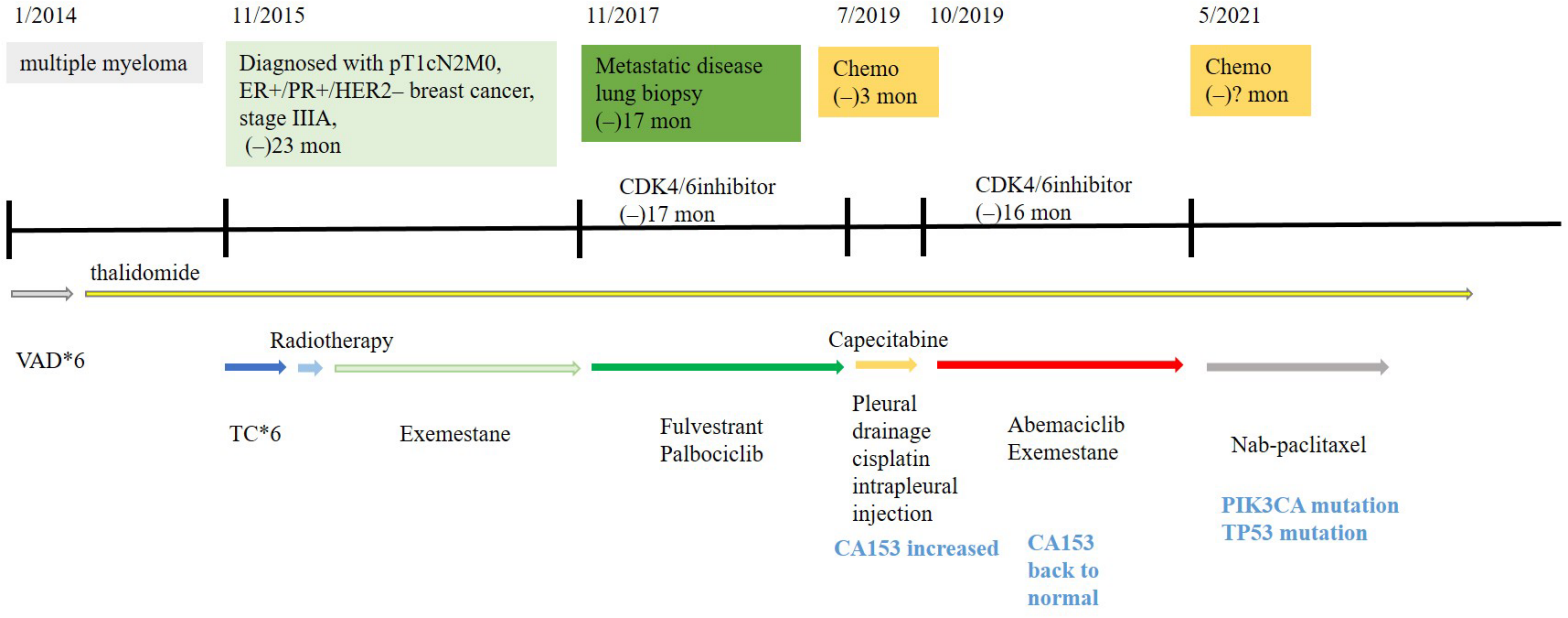
Treatment history of this HR+/HER2− breast cancer patient receiving abemaciclib combined with exemestane after prior progression on palbociclib and fulvestrant.

**Figure 2:**
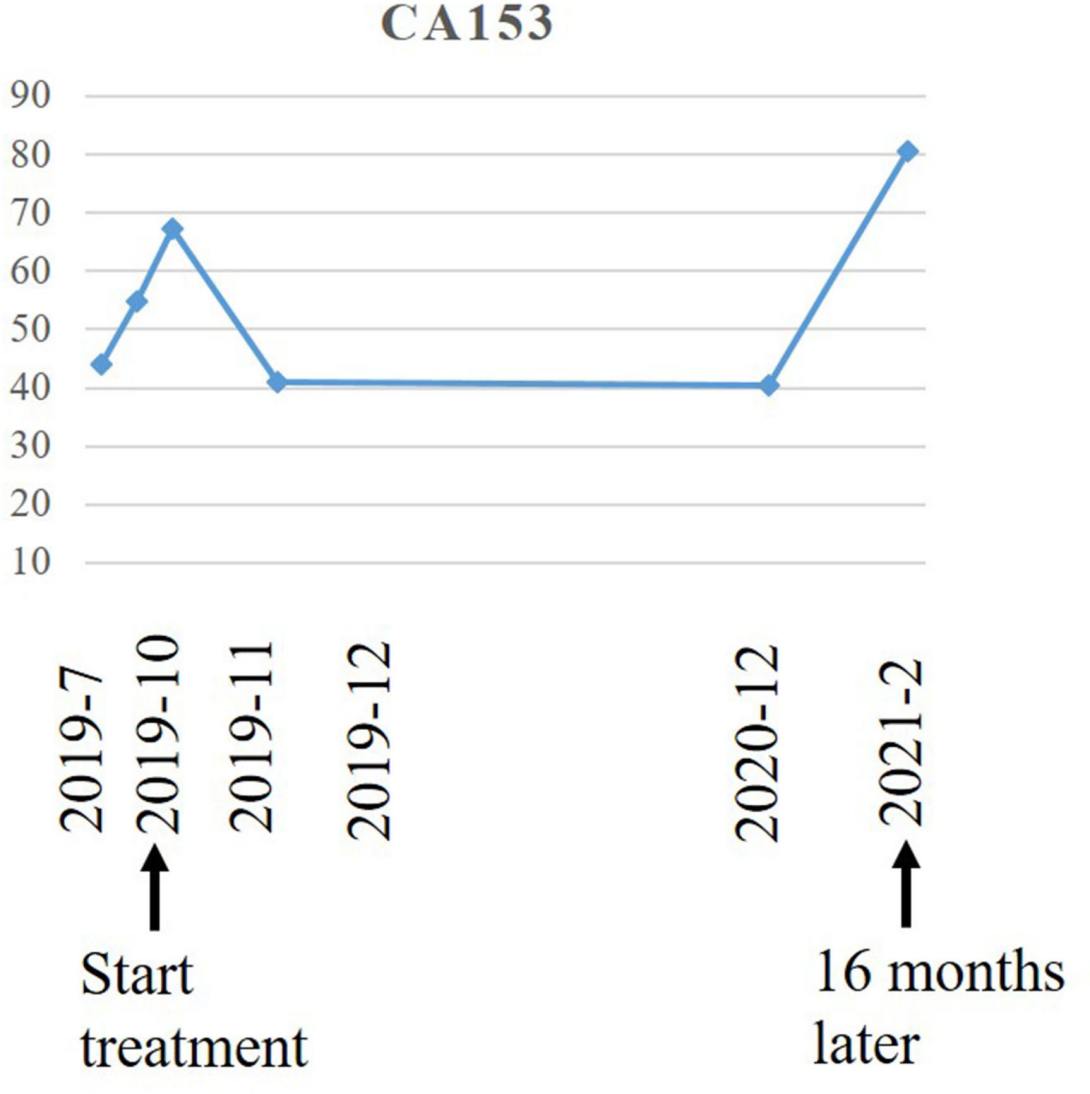
The changes in serum CA153 (U/ml) during the treatment of abemaciclib and exemestane.

**Figure 3:**
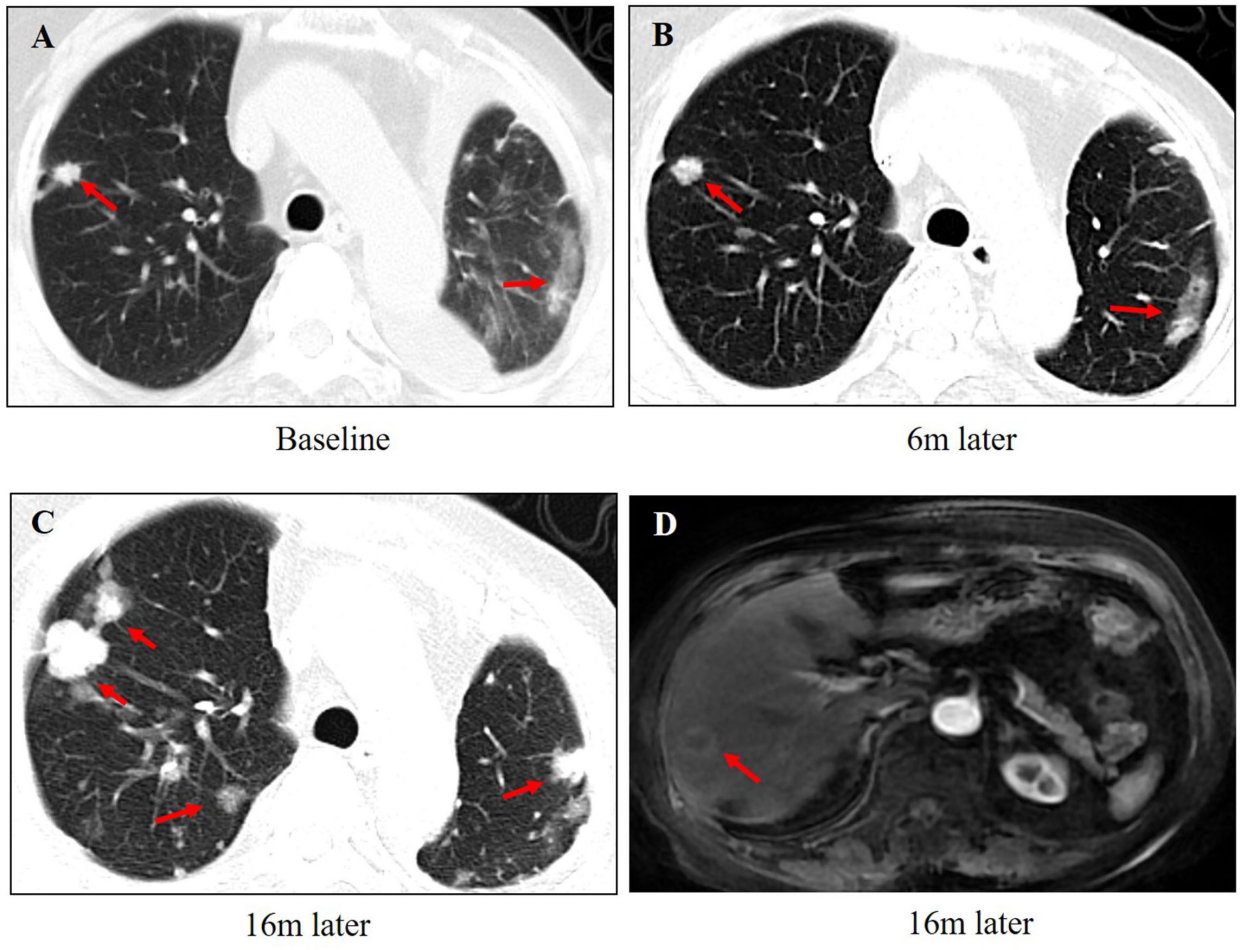
The changes in images during the treatment of abemaciclib and exemestane. **(A)** The baseline of lung metastasis at the beginning of abemaciclib and exemestane. **(B)** The lung lesions were stable after 6 months treatment. **(C)** The lung lesions progressed after 16 months of treatment. **(D)** Liver metastasis was found after 16 months of treatment.

A 65-year-old woman underwent modified radical mastectomy on 17 November 2015. Her treatment is summarized in **Figure 1**. She was diagnosed with invasive ductal carcinoma (histological grade II, tumor size 1.4 × 1 × 1 cm), with 9 out of 18 axillary lymph nodes involved. Pathological stage was pT1cN2M0, stage IIIA. Immunohistochemical (IHC) results showed the following: ER (+++), 80%; PR (+++), 35%; CerbB-2 (0); Ki67 positive rate, 20%; and D2-40, vascular tumor thrombus (+). The patient received six cycles of TC regimen (docetaxel and cyclophosphamide), radiation therapy of the chest wall and regional nodes (50Gy in 25 fractions), and exemestane as adjuvant therapy. Although non-steroidal aromatase inhibitors are, in general, the first choice of adjuvant therapy, only exemestane was reimbursed in our hospital. On 23 November 2017, PET-CT showed multiple small nodules in both lungs, with lung metastases suspected. There were no metastatic signs in other organs. CT-guided lung biopsy showed breast cancer lung metastasis. No further IHC examination was done due to a lack of biopsy tissue. Because disease-free survival (DFS) with endocrine therapy was only 16 months (<24 months) and because she had no visceral crisis, endocrine therapy was considered suitable for her treatment. Fulvestrant was an option. Based on the results of the PALOMA-3 trial, fulvestrant and palbociclib were given as her first line of treatment, and her disease remained stable until May 2019. PFS was 17 months. Grade 2 neutropenia occurred after 2 weeks of palbociclib, with adverse effects reversed after 7 days of palbociclib withdrawal. With re-occurrence of grade 2 neutropenia, she received a one dose reduction in palbociclib, with no more side effects observed. Seventeen months later, she developed chest tightness symptoms with increased serum carbohydrate antigen 153 (CA153) levels. Chest CT showed progression in both lungs, with left pleural effusion. Because she refused intravenous chemotherapy, she received capecitabine for 3 months. Unfortunately, left thoracic cavity effusion increased. Pleural drainage and intra-pleural injection of cisplatin improved symptoms. With the failure of capecitabine and refusal of chemotherapy, endocrine therapy was an option for treatment. With knowledge of the MONARCH-1 study, we administered abemaciclib and exemestane as her third line of treatment. After 1 month, CA153 levels decreased to normal (**Figure 2**), and lung lesions were stable. Although she was 71 years of age, second-line CDK4/6 inhibitor treatment toxicities were tolerable, and only grade 2 diarrhea occurred after 7 days of abemaciclib, which gradually improved after oral administration of Imogen and was normal after 40 days. Sixteen months (on 12-02-2021) later, she was short of breath, and chest CT scan showed progression of pulmonary lesions with liver metastatic lesions (**Figure 3**). Nab-paclitaxel (100 mg) was administered on days 1 and 8 with zoledronic acid for bone preservation. As of May 2022, she was in a stable condition.

She was also diagnosed with multiple myeloma in January 2014, when a thoracic vertebral fracture occurred. VAD (vindesine 1 mg, days 1−4; epirubicin 10 mg, days 1−4; dexamethasone 15 mg bid, days 1−14) chemotherapy regimen was given for a total of six cycles, and then, thalidomide, 100 mg, was given orally every night for 3 months followed by removal for 1 month. Thalidomide was tolerated with no side effects, and the myeloma was stable.

In order to find therapeutic targets for this patient, who progressed on abemaciclib after prior progression on palbociclib, we performed NGS of ctDNA (Geneseeq assay) derived from her blood. A PIK3CA p.E545K exon 9 missense mutation and a TP53 p.H214Lfs*33 frame shift mutation in exon 6 were found (**Figure 1**). The plasma tumor mutation burden (TMB) was 4.1 mutations/megabase [mut/Mb].

## Discussion

Recent studies have shown that the combination of a CDK4/6 inhibitor with hormone therapy was the first choice for treatment of HR+/HER2− MBC because this strategy improved survival (2–8). Palbociclib, abemaciclib, and ribociclib are all CDK4/6 inhibitors that provide similar survival data for MBC patients. Abemaciclib is the only effective monotherapy for HR+/HER2− MBC patients (11). Because of the FDA approval sequence, palbociclib was the first CDK4/6 inhibitor for MBC patients. The MONARCH E study showed abemaciclib to also be effective for high-risk early breast cancer patients (9). With the approval of these drugs, oncologists were challenged to determine the best treatment for HR+/HER2− MBC patients who had failed previous therapy with a CDK4/6 inhibitor.

In this study, we reported outcomes for a HR+/HER2- old MBC patient with a good response to non-sequential abemaciclib and exemestane after fulvestrant and palbociclib treatment failure. The PFS with abemaciclib and exemestane was 16 months for this patient, which was longer than previously reported (10). For MONARCH I and the retrospective multicenter experience, the median PFS for abemaciclib was <8.4 months (10, 11) and only 3.9 months (95% CI, 2.9−5.7, p=.0013) for those who received non-sequential CDK4/6 inhibitor therapy (10). There maybe two reasons. One is that this patient reported herein also had multiple myeloma and was given thalidomide. There are many studies that have shown thalidomide to be immunomodulatory, to have anti-angiogenic activities that may suppress tumor growth (12–14), and to play an important role in cancer control. One recent study found that adding thalidomide to pyrotinib increased clinical benefit for advanced non-small-cell lung cancer (NSCLC) patients with HER2 exon 20 insertions, with reductions in the incidence of pyrotinib-related diarrhea (15). In a breast cancer murine model, Yang Jin et al. found that thalidomide inhibited breast tumor growth through inhibition of angiogenesis by reducing tumor-associated macrophage accumulation and infiltration and decreasing angiogenesis-related cytokine production (14). They also found that thalidomide increased tumor perfusion and decreased vascular leakiness, which may enhance the delivery and efficacy of chemotherapy (12). Meta-analysis showed that thalidomide reduced nausea and vomiting in delayed and overall phases (16), increasing treatment tolerance for older patients. Whether thalidomide can enhance the anti-tumor effect of CDK4/6 inhibitors requires further investigation. The other explanation that this patient had longer PFS may due to the combination of exemestane. Although her BMI was normal (BMI=21) and none of the three aromatase inhibitors (non-steroidal aromatase inhibitors: anastrozole, letrozole, and steroidal aromatase inhibitor exemestane) were superior to the others in terms of efficacy and safety, some studies still indicated that exemestane can significantly decrease serum levels of leptin while letrozole cannot (17). This hypothesis also needs to be further verified.

The patient reported herein was older, refused chemotherapy, and was satisfied with her two lines of endocrine therapy, which were well tolerated and provided lasting tumor control.

Previous studies found that the PIK3CA mutation, ERS1 mutation, cyclin E1 (CCNE1) amplification, and the CCND1 amplification contributed to endocrine resistance (18–23). The exploratory analysis of the PALOMA-3 study showed that high CCNE1 mRNA expression was associated with the poor anti-tumor activity of palbociclib (18). Lee et al. found that high TMB, the TP53 mutation, the PTEN loss of function mutation, and RB1 pathway alteration related to palbociclib resistance (19). Gene expression analysis of baseline tumor mRNA by the MONALEESA-7 study found survival benefit for patients treated with ribociclib who had high expression levels of CCND1, IGF1R, and ERBB3, and for patients with low expression levels of CCNE1 and MYC (21). A retrospective analysis found that RB1, ERBB2, and CCNE1 alterations contributed to rapid cancer progression with abemaciclib (10). There are barriers and limitations to the use of NGS for metastatic cancer patients. Many trials have used NGS to identify biomarkers and new drug targets for treatment of breast cancer patients (18, 21). Anna et al. found that for HR+/HER2− MBC patients with the BRCA mutation, a combination of PARP inhibitors, palbociclib, and letrozole was the most effective cancer treatment (24). Wang et al. found heavily pre-treated HR+/HER2− MBC patients with high TMB to respond well to camrelizumab (25). These studies provide insight into new strategies by which to treat patients who were previously treated with many lines of endocrine and/or chemotherapy. In this case report, we used a new approach for the treatment of this patient. Because the patient denied biopsy of her new metastatic lesions, we collected a blood sample and, by NGS, found a PIK3CA p.E545K exon 9 missense mutation and a TP53 p.H214Lfs*33 frame shift mutation in exon 6. The TMB in plasma was 4.1 mutations/megabase (mut/Mb), which was quite low. Many studies have demonstrated the PIK3CA mutation to play an important role in endocrine resistance. PIK3CA-mutated HR+/HER2− MBC cells are less sensitive to chemotherapy (26), which may explain why this patient showed no response to capecitabine. Although the TP53 mutation is common in MBC patients, there are no target drugs for breast cancer patients. However, PIK3CA mutations have predictive value for treatment with the α-selective PI3K inhibitor, alpelisib, and the β-sparing PI3K inhibitor, taselisib (SANDPIPER trial), in an advanced situation (26, 27). Based on the NGS results, this patient may be sensitive to PI3K/AKT/mTOR pathway inhibitors. Considering drug accessibility and tolerance, she finally received nab-paclitaxel, 100 mg, on days 1 and 8 and was in a stable condition as of May 2022.

## Conclusion

In conclusion, we reported an HR+/HER2− MBC patient, also diagnosed with multiple myeloma, who showed a good response to non-sequential abemaciclib and endocrine therapy after cancer progression following palbociclib therapy. The PFS of abemaciclib for this patient was longer than that reported previously. Furthermore, ctDNA plasma sequencing of this patient showed PIK3CA and TP53 mutations, which indicated that PI3K inhibitors may be one option for her future treatment. Overall, these findings suggested that some patients may benefit from continued CDK4/6-directed therapy, even though the utility of individual CDK4/6 inhibitors is unknown. Thus, additional, large-sample, prospective trials are necessary to evaluate the effectiveness of CDK4/6 inhibitors in such clinical situations.

## Data availability statement

The datasets presented in this study can be found in online repositories. The names of the repository/repositories and accession number(s) can be found in the article/Supplementary material.

## Ethics statement

The studies involving human participants were reviewed and approved by the ethics committee of the Affiliated Hospital of Qingdao University (QYFY WZLL 27231). The patients/participants provided their written informed consent to participate in this study.

## Author contributions

YM contributed to the conception and writing of the manuscript. ML and YW were involved in explaining the data and doing the figures. WC interpreted the radiological images. WL contributed to the NGS test and reviewed the manuscript. All authors reviewed and approved the final version of this manuscript.
